# Supplementary material for: Fermi-Surface Topological Phase Transition and Horizontal Order-Parameter Nodes in CaFe2As2 Under Pressure
Source: Sci Rep. 2016 May 24;6:26394. doi: 10.1038/srep26394 (PMC4877643; doi:10.1038/srep26394)
Supplement: Supplementary Information [file srep26394-s1.pdf]

# Supplementary Information

# Fermi-surface topological phase transition and horizontal order-parameter nodes in $CaFe_2As_2$ under pressure

R. S. Gonnelli,[1] D. Daghero,[1] M. Tortello,[1] G. A. Ummarino,[1] Z. Bukowski,[2] J. Karpinski,[3] P. G. Reuvekamp,[4] R. K. Kremer,[4] G. Profeta,[5] K. Suzuki,[6] and K. Kuroki[6]

[1]*Dipartimento di Scienza Applicata e Tecnologia, Politecnico di Torino, 10129 Italy*
[2]*Polish Academy of Sciences, 50-950 Wrocław, Poland*
[3]*Ecole Polytechnique Fédérale de Lausanne, CH-1015 Lausanne, Switzerland*
[4]*Max Planck Institute for Solid State Research, Stuttgart, Germany*
[5]*Dipartimento di Scienze Fisiche e Chimiche, Università dell'Aquila, L'Aquila, Italy*
[6]*Department of Physics, Osaka University, Toyonaka, Osaka 560-0043, Japan*

## ERROR BARS AND ROBUSTNESS OF THE FITS

In Fig. 6 we showed the *best* fit of the low-temperature PCARS spectra, obtained by minimizing the sum of squared residuals (SSR). This corresponds to minimizing the chi-square, but does not require an estimation of the uncertainty of the conductance for each point, which can vary from curve to curve and is often difficult to estimate [S1]. The same holds for the data points that represent the gap values in Fig. 8. However, in the same figure each gap value has a vertical error bar that represents the range of possible gap values that give acceptable fits to a given experimental spectrum. This range was evaluated as follows:

1. first, we decide a confidence band for the fits; this means that we fix a priori the maximum SSR that can be accepted for a reasonable fit. Usually it is of the order of two or three times the minimum one;
2. then we progressively increase (decrease) one of the two gaps, and try to fit the curve –keeping the SSR in the fixed confidence band– by changing the other fitting parameters (not all of them must be necessarily changed, as shown in the following); then we repeat the procedure with the second gap;
3. thus, we obtain a range of gap values that give a fit with a SSR inside the confidence band *when all the other parameters are changed as well*. This range is represented as an error bar in Fig. 8.

In order to clarify this procedure with an example, in Figure S1 we show the effect of a controlled modification of the value of the small gap (Fig. S1a) and of the large gap (Fig. S1b) on the other fitting parameters, with the constraint that $SSR < 3 \cdot SSR_{best}$. As a starting point we used the best-fitting parameters of the curve shown in Fig. 6c of the paper ($P = 0.61$ GPa, $T = 1.27$ K) that are represented by green circles in the graphs. Then we varied by $\pm 10\%$ (orange and cyan squares) and by $\pm 20\%$ (red and violet triangles) the gap values adjusting all the other fitting parameters in order to obtain a good fit with $SSR < 3 \cdot SSR_{best}$.

Figure S1a clearly shows that the increase/decrease in $\Delta_h$ corresponds only to a proportional increase/decrease in $\Gamma_h$, and to a decrease/increase in $Z_h$. The parameters of the other band are only slightly adjusted. Quite a similar situation occurs for the increase/decrease of $\Delta_e$ (Figure S1b). It is worthwhile to mention that all the fitting parameters represented by the same colored symbol in Fig. S1 produce an acceptable fit with a SSR quite close to the best one. This proves the robustness of the fits, of course within a certain level of uncertainty that is just what we represented by the error bars in Fig. 8 of the main text.

## DEPENDENCE OF THE RESULTS ON THE FERMI SURFACE TOPOLOGY AND ORDER PARAMETER SYMMETRY

As explained in the main text, DFT and RPA calculations provided a clear indication of the FS shape and of the most likely OP symmetry in the T* phase, and we used the relevant results for the 3D-BTK fit. However, it is interesting to discuss here what happens to the fit when the shape of the FS and the OP symmetry are changed.

Actually, the range of possible FS geometries and OP structures is strongly limited by the shape of the experimental PCARS spectra. It has been shown elsewhere [S2, S3] and mentioned in the main text that the occurrence of the

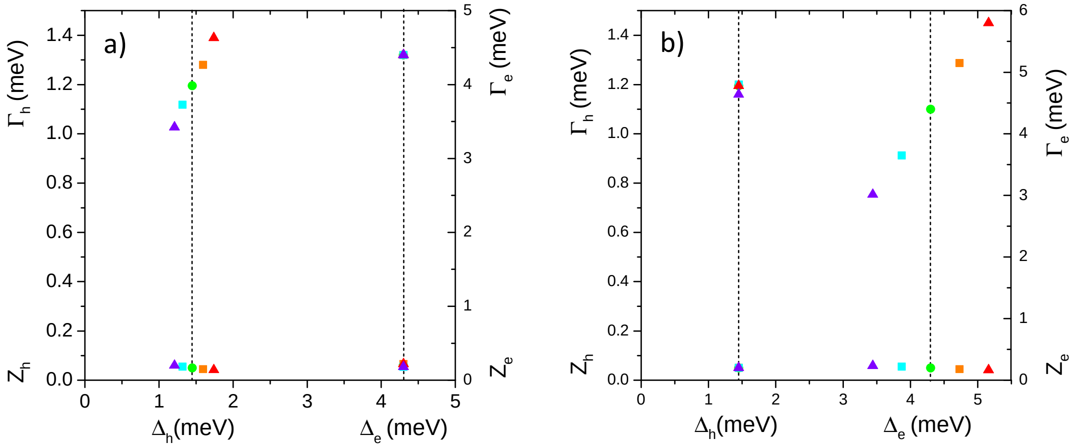


FIG. S1. The effect of a controlled modification ($\pm10\%$ $\rightarrow$ orange and cyan squares, $\pm20\%$ $\rightarrow$ red and violet triangles) of the best value of the small gap (green circles in panel a) and of the large one (green circles in panel b) on the other fitting parameters maintaining a good fit with SSR $<$ 3$\cdot$ SSR$_{\text{best}}$. The procedure is applied to the curve shown in Fig. 6c of the main text ($P = 0.61$ GPa, $T = 1.27$ K).

zero-bias conductance maximum in both the *ab*-plane and the *c*-axis spectra can be explained within the 3D BTK model only if a small and strongly anisotropic gap (with nodes, zeros or, at least, deep minima) resides on a FS sheet with a marked 3D character (i.e. *not* similar to an hyperboloid, but rather to a spheroid). A larger gap, possibly isotropic (or, at least, with a small anisotropy) is necessary as well to explain the shoulders at finite energy. The 3D BTK model alone, however, cannot be used to predict the shape of the FS that carries the large gap or the relative size of the FS sheets. DFT calculations in the T* phase tell us that the quasi-3D FS sheet is the holelike one, and also provide the missing quantitative information about both the FS sheets. However, since we used in the 3D BTK model a simplified FS that captures all the essential physics from the point of view of Andreev reflection, the tiny details of the real FS are unimportant. For example, the presence of a cylindrical neck along the Γ-Z direction and its size are not crucial for obtaining a good fit. However, we know that when the neck is absent (i.e. in the cT phase) or too wide (i.e. in the OR phase) the system is not superconducting. Thus, the FS used in the 3D BTK model is, in practice, the only possible choice – and, at the same time, the most general one.

As for the $k$ dependence of the OP, once the possible small in-plane anisotropy of $\Delta_e$ is neglected for simplicity, the only degree of freedom left is the shape of the lines of nodes (or zeros) on the quasi-3D holelike FS (i.e. the $k$ dependence of $\Delta_h$). Let us start from the best fits shown in Fig. 6c and 6e of the paper, which we choose as being representative of *ab*-plane and *c*-axis contacts, and see what happens if a different symmetry of $\Delta_h$ (with respect to that used in the main text) is assumed. For simplicity, and following the theoretical constraints for the possible gap structures in $CaFe_2As_2$, we consider only three alternative cases, i.e.:

1. $\Delta_h$ has the same in-plane anisotropy described in the paper, but features a horizontal node line at $\phi = 17\pi/18$ (where the cylindrical neck of the FS is connected to the cup-shaped part) rather than at $2\pi/3$ (as in the main text and in the RPA calculations). In this way, there is no change of the sign of the OP on the cup-like FS (see Fig. S2a). This is done in order to check the effect of the vertical displacement of the horizontal node on the fitting results;

2. $\Delta_h$ has 4 vertical lines of zeros but no dependence on the polar angle $\phi$, i.e. no horizontal nodes (see Fig. S2b);

3. $\Delta_h$ has 4 vertical lines of *minima* (ratio max/min = 3), but no dependence on the polar angle $\phi$ (see Fig. S2c). In practice this in-plane anisotropy is the same as in the paper, but with the polar dependence removed.

The results of the fits of the experimental curves of Fig. 6c (*ab*-plane) and 6e (*c*-axis) by using the symmetries for $\Delta_h$ shown in Fig. S2 a, b and c can be summarized as follows:

In principle, and from the pure technical point of view, it is possible to fit both the *ab*-plane and the *c*-axis experimental curves by using a symmetry for the small gap $\Delta_h$ with a horizontal node (at any $k_z$ level) or with 4 vertical lines of zeros or with 4 vertical lines of deep minima. However, moving from the first to the second and the third symmetry, the fit requires strongly decreasing values of $\Delta_h$ (that in the worst case is only about one half of the original value) and a strong increase of the ratio $\Gamma_h/\Delta_h^{max}$ (that in the worst case, along the *c*-axis direction, exceeds 2.2). These two effects, that are due to the change of the OP symmetry with respect to the fits shown in

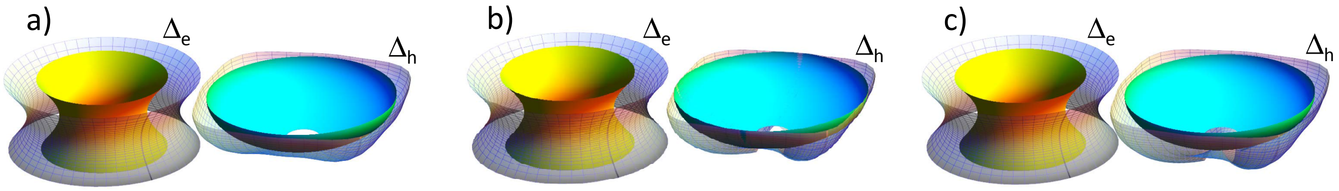


FIG. S2. The three different symmetries for the small order parameter on the hole FS ($\Delta_h$) used in the fitting tests performed on the curves shown in Fig. 6c and 6e. In particular: a) $\Delta_h$ with the same in-plane anisotropy described in the main text, but with a horizontal node line at $\phi = 17\pi/18$; b) $\Delta_h$ with 4 vertical lines of zeros, but no dependence on the polar angle $\phi$; c) $\Delta_h$ with 4 vertical lines of minima, but no dependence on the polar angle $\phi$.

the main text, are negative facts both from the physical and the technical point of view. Indeed, the strong decrease of $\Delta_h$, especially for the symmetries (2) (Fig. S2b) and (3) (Fig. S2c) leads, in particular for $c$-axis curves, to a ratio $\Delta_e/\Delta_h^{max}$ that far exceeds the values (between 2 and 3.5) usually observed in all the experiments on Fe-based compounds. Additionally, the ratio $\Gamma_h/\Delta_h^{max}$ (again particularly along the $c$-axis direction) ends up by exceeding the maximum accepted value for a reliable fit (of the order of 1).

Thus we can conclude that our experimental results and the fits, *particularly those in c-axis contacts*, tell us that:

1) *The hole FS must have a cup-shaped geometry around the Z point.* In principle the presence of a cylindrical neck around the Γ point and its dimensions are not crucial for obtaining a good fit;

2) *A symmetry of the order parameter on the hole FS with a horizontal node line gives*: i) the best fit, i.e. the minimum SSR; ii) a ratio $\Delta_e/\Delta_h^{max}$ in agreement with all the other measurements in Fe-based superconductors; iii) a ratio $\Gamma_h/\Delta_h^{max}$ smaller or close to 1; iv) last but not least, a complete agreement with the results of ab-initio RPA calculations independently obtained starting from the FS topology calculated in the T* phase.

[S1] D. Daghero et al., J. Phys.: Condens. Matter **20**, 085225 (2008).
[S2] D. Daghero, M. Tortello, G.A. Ummarino and R.S. Gonnelli, Rep. Prog. Phys. **74**, 124509 (2011).
[S3] R. S. Gonnelli et al., Supercond. Sci. Technol. **25**, 065007 (2012).
